# Transfrenular Single-Incision (TrSI) Flaps: A Modified Minimally Invasive Technique That Optimizes Aesthetic Outcomes in Maxillary Central Incisor Apicoectomy—Technique Description and Case Report

**DOI:** 10.3390/dj14080507

**Published:** 2026-08-10

**Authors:** Ivan Hristov Arabadzhiev, Eber Luis Lima Steolo, Carsten Nix

**Affiliations:** 1 Oral Surgeon, Private Practice, 66849 Landstuhl, Germany; carsten-nix@gmx.de; 2 Oral and Maxillofacial Surgeon, Private Practice, Pato Branco 80501-054, PR, Brazil; drebersteolo@gmail.com

**Keywords:** transfrenular flap, apicoectomy, maxillary aesthetic flap, Eskici vertical flap, paraendodontic surgery

## Abstract

**Background and Objectives**: Achieving optimal soft-tissue aesthetics is a paramount challenge during an apicoectomy of the maxillary central incisors. This article presents a novel modification of the Eskici Vertical Flap, designed to minimize scar formation, preserve the architectural integrity of the sub-mucosal fibers, and eliminate the risk of marginal gingival recession. **Methods**: The Transfrenular-Single Incision (TrSI) flap is elevated via a precise two-step process. First, a sagittal incision is carried out through the mucosa along the midline of the maxillary labial frenulum. Blunt lateral tunnel preparation is then performed toward the targeted tooth apex, and a subsequent deep periosteal incision with bony contact provides localized access to the periapical lesion. Following Paraendodontic Surgical Intervention (PSI), a two-layer suturing technique is implemented: the deep layer utilizes resorbable sutures to restore periosteal integrity and reposition the basal fibers of the frenular connective tissue, while the superficial layer promotes accurate mucosal edge adaptation. **Results**: The surgical access provided by the TrSI flap provides clinically sufficient exposure to perform precise osteotomy, retrograde root-end preparation, and biocompatible sealing. The evaluated clinical cases demonstrated optimal healing kinetics, the complete absence of visible scar tissue, and zero marginal gingival recession. **Conclusions**: The TrSI flap modification constitutes a highly predictable and effective option for PSI in the aesthetically critical anterior maxilla. Although its application spectrum is primarily limited to the central incisors, its capacity to prevent aesthetic deformities warrants its consideration in selected surgical protocols.

## 1. Introduction

Paraendodontic Surgical Intervention (PSI), or simply apicoectomy, is a well-established modality in oral surgery dating back to the late 19th century. Despite continuous advancements in endodontic materials and instruments, primary orthograde root canal treatments and conventional retreatments occasionally fail to completely heal periapical lesions.

In a comprehensive meta-analysis, Mushtaq et al. [1] reported a success rate of up to 92.0% at 24 months following primary endodontic treatment, which decreased to 83.5% in retreatment cases; beyond 3 years, the success rate further declined to 73.7%. Consequently, surgical root-end intervention represents the logical sequential step when orthograde alternatives are exhausted. Modern PSI exhibits favorable survival rates of 93.7% at 2 years, 90.5% at 4 years, and 88.0% at 6 years [2]. While exact longitudinal survival rates vary across the literature, the integration of microsurgical magnification and biocompatible retrograde filling materials provides an excellent long-term prognosis.

The objective of modern PSI extends beyond tooth preservation; it encompasses meticulously preserving the adjacent periodontal soft tissues. Consequently, various flap designs have been developed to achieve this balance. Grandi and Pacifici [3] categorized 10 distinct flap designs used for endodontic surgical access, concluding that no universal, optimal flap design exists regarding cosmetic outcomes. Their findings emphasize that a carefully tailored approach, guided by rigorous soft-tissue management, is mandatory to prevent aesthetic impairment and wound dehiscence.

This paper introduces the Transfrenular Single Incision (TrSI) flap. This technique is classified as a single-incision flap based on Eskici’s [4] modified flap for PSI. The authors developed and optimized this modified single-incision technique specifically for the apicoectomy of maxillary central incisors to maximize aesthetic outcomes in the anterior “smile zone.” The clinical cases presented herein demonstrate excellent healing without visible scarring or marginal gingival recession. Additionally, the specialized two-layer closure mechanism, which incorporates the submucosal fibers of the frenulum during retraction and subsequent repositioning, provides a stable, tight bacterial seal that prevents soft-tissue dehiscence.

## 2. Materials and Methods

The Transfrenular Single-Incision (TrSI) technique is executed through a precise, minimally invasive sequence designed to isolate the deep bone-handling phases from superficial mucosal aesthetics. This approach relies on a specialized, two-layer surgical framework where the initial mucosal window and the subsequent deep periosteal access are laterally offset, with the detailed step-by-step protocol outlined sequentially below.

### 2.1. Detailed Description of the Surgical Technique

Step 1 (Primary Mucosal Incision): Under local anesthesia, a sharp incision is made precisely along the sagittal midline of the maxillary labial frenulum using a conventional No. 15 scalpel blade, with incision length adapted to the patient’s anatomy, utilizing the full extent of the frenulum from the most coronally attached fibers up to the vestibular fold. This initial incision must penetrate deep into the mucosal and submucosal layers to reach the periosteum without cutting through it (avoiding direct bone contact).

Step 2 (Blunt Tunnel Dissection): Fine, blunt dissecting scissors are introduced into the incision to prepare a lateral submucosal tunnel directed toward the periapical region of the targeted central incisor. This tunnel is developed through the lateral fibers of the frenulum, extending over the root apex. The dissection is strictly bound by the mucosa ventrally and the intact periosteum dorsally, and no sharp scalpel dissection is performed during this phase.

Step 3 (Secondary Periosteal Incision): Once adequate tunnel volume is established over the root of the first incisor, a second, sharp vertical incision is performed directly through the periosteal tissue down to the cortical bone, with this periosteal incision positioned approximately 2–3 mm mesial to the root midline.

Step 4 (Periosteal Elevation): A periosteal elevator is inserted through the tunnel to reflect the periosteal tissue laterally, exposing the underlying cortical plate and the periapical bone defect.

Step 5 (Retraction and Endodontic Intervention): A small Langenbeck retractor (Ustomed; Tuttlingen, Germany) is placed into the tunnel to maintain clear visualization. Then, standard osteotomy, root-end resection (apicoectomy), retrograde cavity preparation, and biocompatible sealing are performed.

Step 6 (Deep and Superficial Layers Closure): The periosteum is repositioned and closed independently using 5-0 resorbable sutures (SABAsorb rapid, Sabana Medizinbedarf, Wiesbaden, Germany). This primary layer is used to reconstruct the architectural integrity of the periosteum and accurately reposition the basal connective tissue fibers of the frenulum. The mucosal edges are adapted using 5-0 non-resorbable sutures (SABApol, Sabana Medizinbedarf, Wiesbaden, Germany) to completely reconstruct the labial frenulum’s anatomical form, and non-resorbable mucosal sutures are removed after a 7-day healing period.

### 2.2. Graphical Presentation of Surgical Steps in Frontal and Axial View

**Figure 1:**
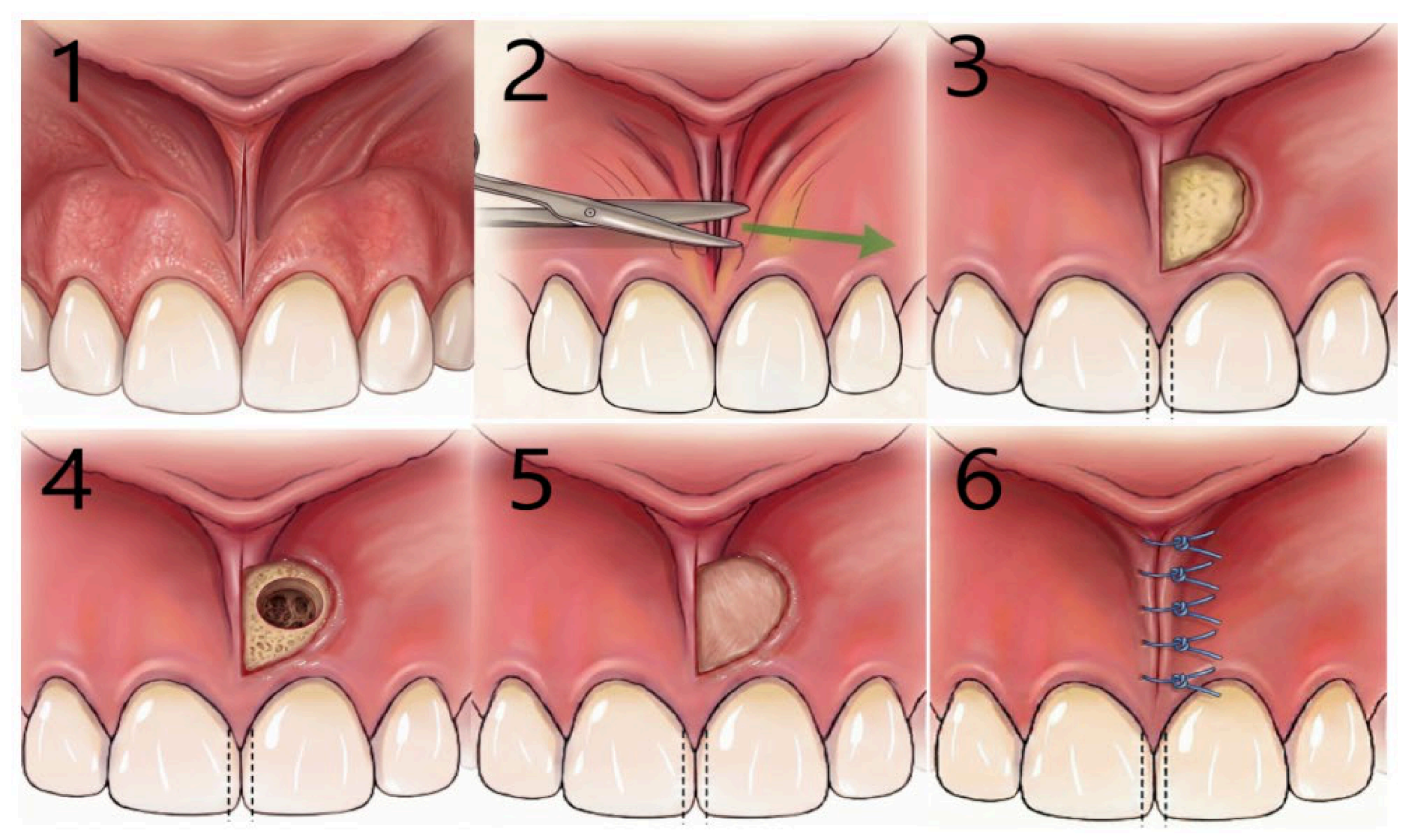

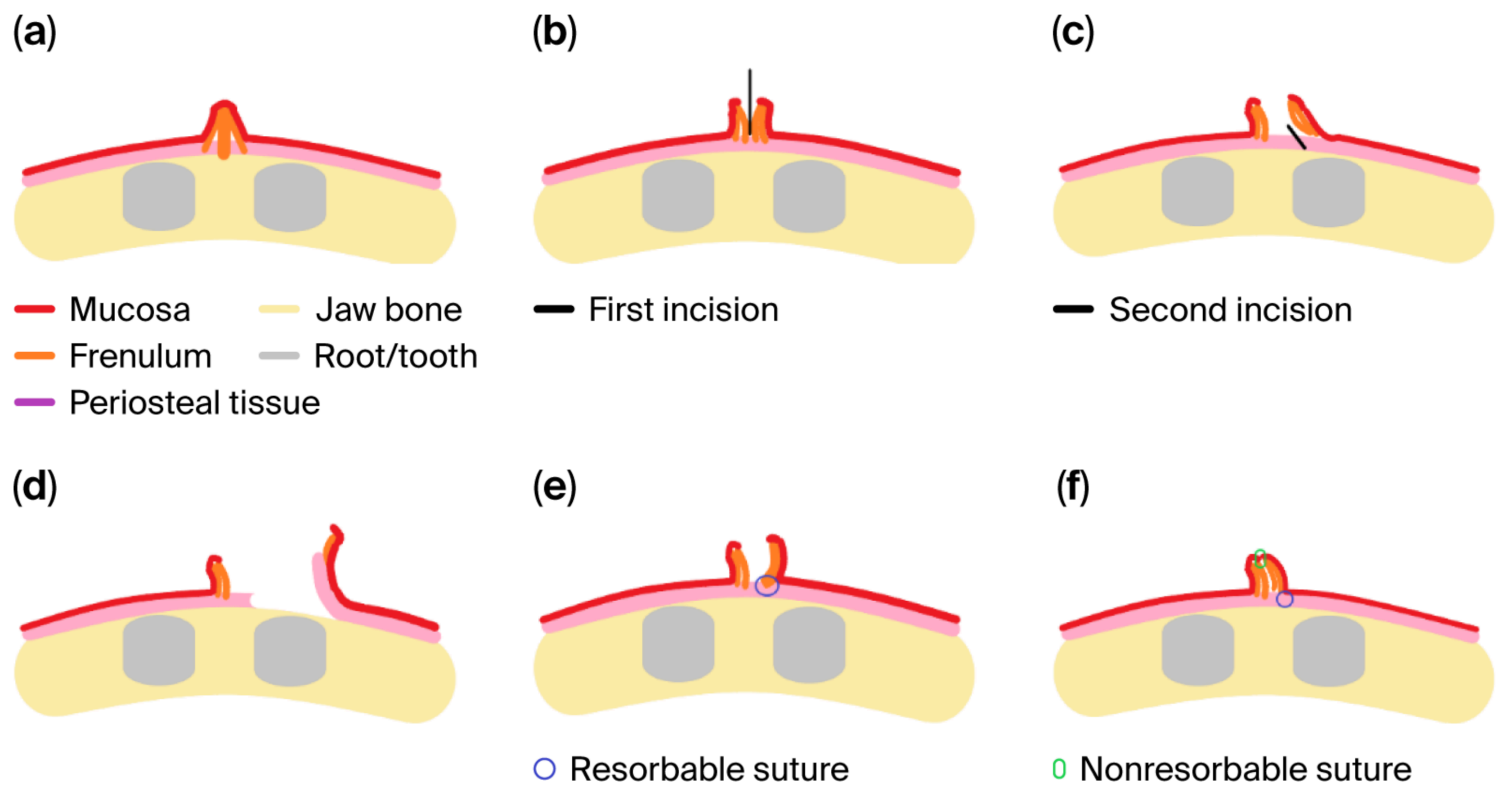
Schematic diagram illustrating the sequential steps of the TrSI technique: Frontal View: (**1**) Sagittal midline mucosal incision within the labial frenulum; (**2**) blunt scissor-guided lateral tunnel dissection; (**3**) periosteal elevation and cortical bone exposure; (**4**) periapical osteotomy execution; (**5**) deep-layer periosteal repositioning and resorbable suturing; (**6**) final deep and superficial mucosal closure along with frenulum anatomical reconstruction. Axial View: (**a**) the anatomy prior to the surgery; (**b**) initial incision through the frenulum; (**c**) the second periosteal incision; (**d**) the fully retracted flap; (**e**) frenulum reconstruction; (**f**) the final superficial closure of the frenulum and mucosa.

Figure 1 presents the TrSI technique in axial view. Image “(a)” presents the anatomy prior to the surgery. The red color outlines the mucosa, while the orange and purple lines depict the frenulum fibers and periosteal tissue, respectively. The schematic image for the teeth is shown in gray. Image “(b)” represents the initial incision through the frenulum. Step “(c)” marks the position of the second periosteal incision lateral from the middle line. The fourth image, “(d)”, shows the fully retracted flap and the free bony surface vestibular to the root. “(e)” highlights periosteal integrity, with the base of the frenulum reconstructed with resorbable sutures. Lastly, step “(f)” represents the final superficial closure of the frenulum fibers and the mucosa with non-resorbable sutures.

## 3. Case Report and Results

### 3.1. Case 1

A 58-year-old female patient was referred to our oral surgery clinic for an indicated apicoectomy of the maxillary left central incisor. The patient reported a history of recurrent pain and swelling in the affected region. The patient’s medical history indicated type 2 diabetes mellitus as a relevant systemic risk factor, which was well controlled with metformin. Following a comprehensive clinical intraoral evaluation and preoperative radiographic diagnostics, the patient was diagnosed with apical periodontitis of the maxillary/mandibular left central incisor. Alternative treatment modalities were discussed, with surgical root-end intervention determined as the appropriate treatment. The surgical protocol was explained in detail, and written informed consent was obtained from the patient.

Prior to surgery, the patient was draped under standard sterile conditions, and preoperative antisepsis was performed using a 0.12% chlorhexidine digluconate mouthwash (Gum Paroex; Sunstar, Schönau, Germany). Local anesthesia was achieved via local infiltration of approximately 1.5 mL of 4% articaine with 1:200,000 adrenaline (Ultracain D-S; Septodont, Frankfurt am Main, Germany).

**Figure 2:**
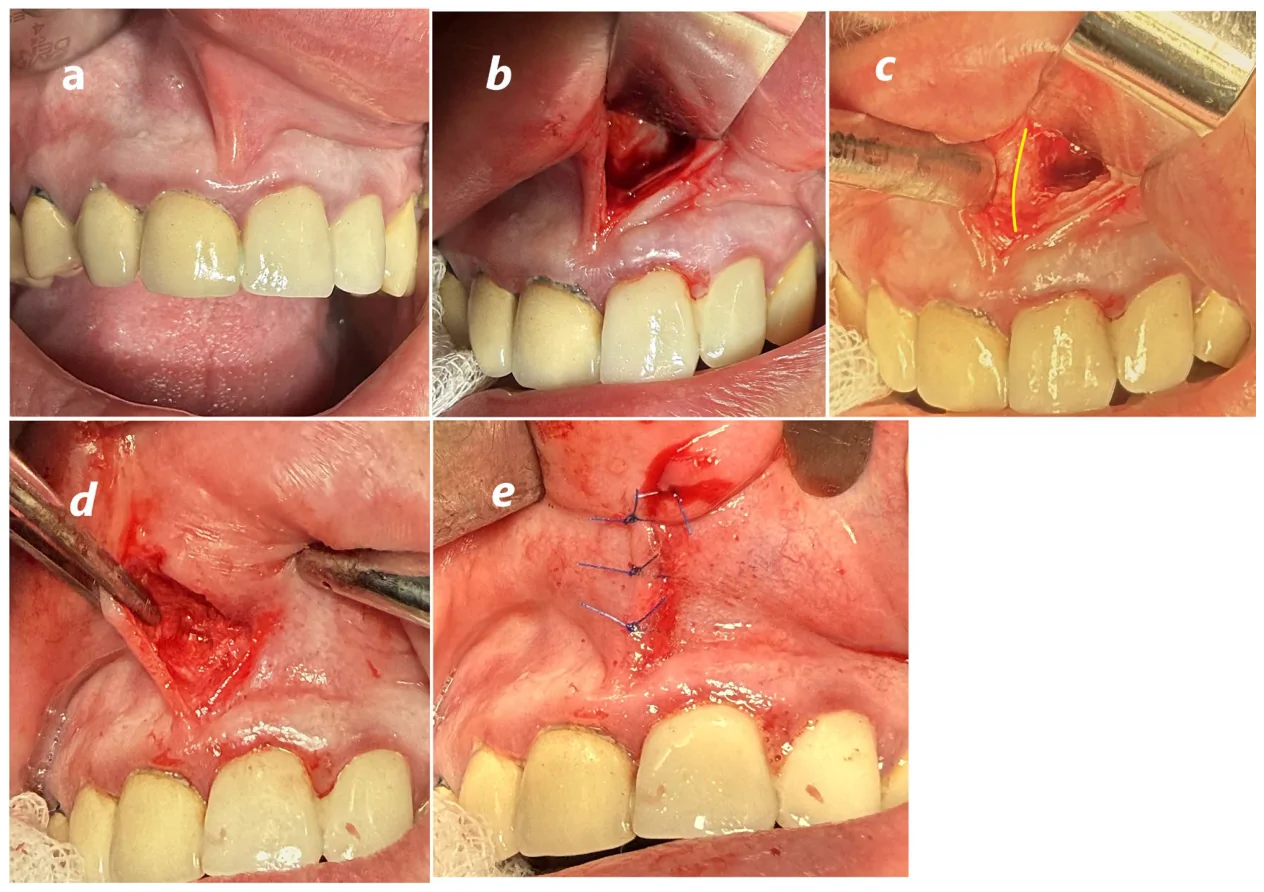
Intraoperative documentation of Case 1: (**a**) preoperative soft-tissue and the labial frenulum’s condition; (**b**) placement of the Langenbeck retractor (Ustomed; Tuttlingen, Germany) through the transfrenular mucosal incision, demonstrating an intact deep periosteal layer; (**c**) elevation and retraction of the periosteal flap showcasing the periapical bone cavity after osteotomy (the yellow line depicts the second incision through the periosteal tissue, with the lateral part of the tissue already retracted); (**d**) repositioning and closure of the deep periosteal layer with 5-0 resorbable sutures (SABAsorb rapid; Sabana Medizinbedarf, Wiesbaden, Germany); (**e**) final superficial mucosal closure using a 5-0 monofilament non-resorbable polypropylene suture (SABApol; Sabana Medizinbedarf, Wiesbaden, Germany).

**Figure 3:**
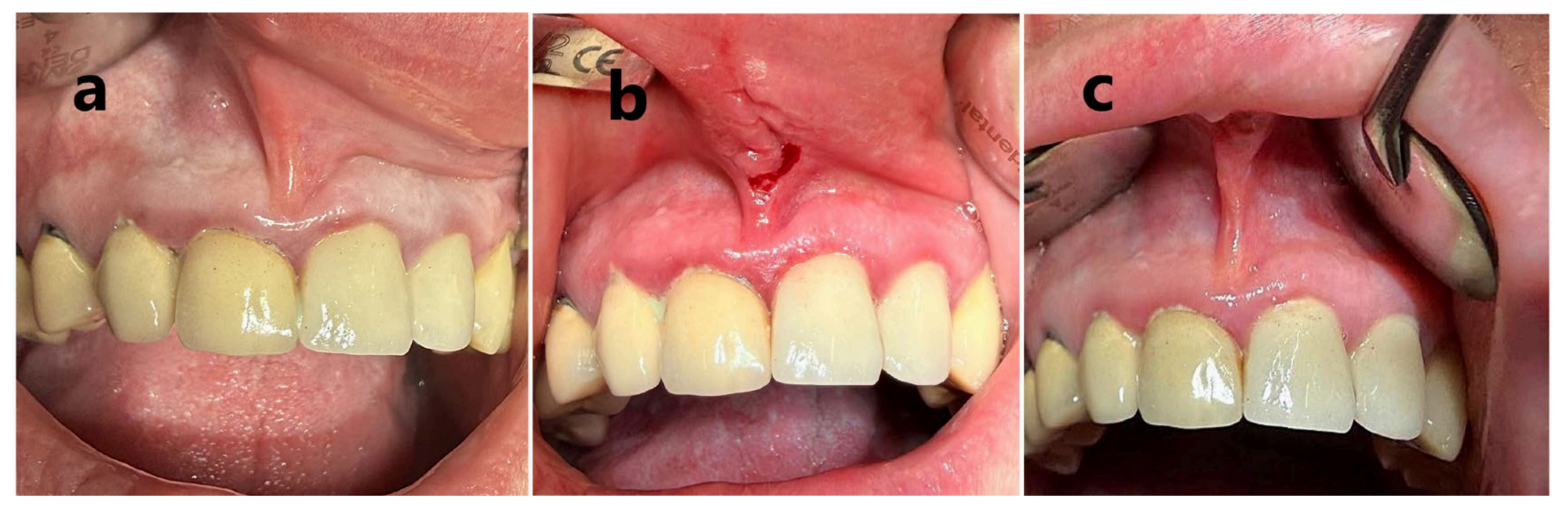
Clinical follow-up sequence of Case 1: (**a**) preoperative baseline presentation; (**b**) soft-tissue healing status on postoperative day 7 immediately following suture removal; (**c**) longitudinal follow-up at 3 months displaying complete soft-tissue rehabilitation, excellent aesthetics, and absent scar formation.

The surgical procedure strictly adhered to the described Transfrenular Single-Incision (TrSI) flap protocol, and the baseline clinical condition of the labial frenulum was thoroughly assessed (Figure 2a and Figure 3a). The primary sagittal midline mucosal incision was performed using a No. 15 scalpel blade (B. Braun; Melsungen, Germany). Following blunt lateral submucosal dissection with fine scissors, a Langenbeck retractor (Ustomed; Tuttlingen, Germany) was carefully inserted through the transfrenular mucosal window, thus maintaining a completely intact deep periosteal layer (Figure 2b).

Subsequently, a secondary vertical incision was made directly through the periosteum, followed by Langenbeck retractor (Ustomed; Tuttlingen, Germany) insertion to facilitate visualization. Then, periapical osteotomy was executed using a round bur H 141 (Komet Dental; Lemgo, Germany), followed by root-end resection with a Lindemann bur (Komet Dental; Lemgo, Germany). Finally, retrograde cavity preparation was performed using a sonic scaler handpiece (SONICflex; KaVo Dental, Biberach, Germany), and the cavity was subsequently sealed with a premixed bioceramic putty (NeoPUTTY; Avalon Biomed, Houston, TX, USA).

Anatomical reconstruction was performed in two separate layers. Specifically, the deep periosteal layer and the basal fibers of the labial frenulum were successfully repositioned and closed independently using a 5-0 monofilament resorbable suture (Serafast; Serag-Wiessner, Naila, Germany) (Figure 2d), while final superficial mucosal adaptation was achieved using a 5-0 monofilament non-resorbable polypropylene suture (SABApol; Sabana, Wiesbaden, Germany), thus completely restoring the original anatomy of the frenulum (Figure 2e).

Postoperative pharmacology consisted of antibiotic amoxicillin (1000 mg, three times daily for 7 days) and anti-inflammatory ibuprofen (400 mg). On the sixth postoperative day, the patient returned for follow-up, and the non-resorbable sutures were removed. Clinical evaluation immediately after suture removal demonstrated excellent soft-tissue healing kinetics (Figure 3b), and longitudinal clinical control at the 3-month follow-up confirmed complete soft-tissue rehabilitation, exceptional “red aesthetics,” and an absolute absence of marginal gingival recession or visible scar formation (Figure 3c).

Upon suture removal, the patient reported no pain and only mild discomfort during mastication. At the 3-month follow-up, the patient remained completely asymptomatic with no reported pain or discomfort, expressing high satisfaction with both the functional and aesthetic outcomes.

### 3.2. Case 2

**Figure 4:**
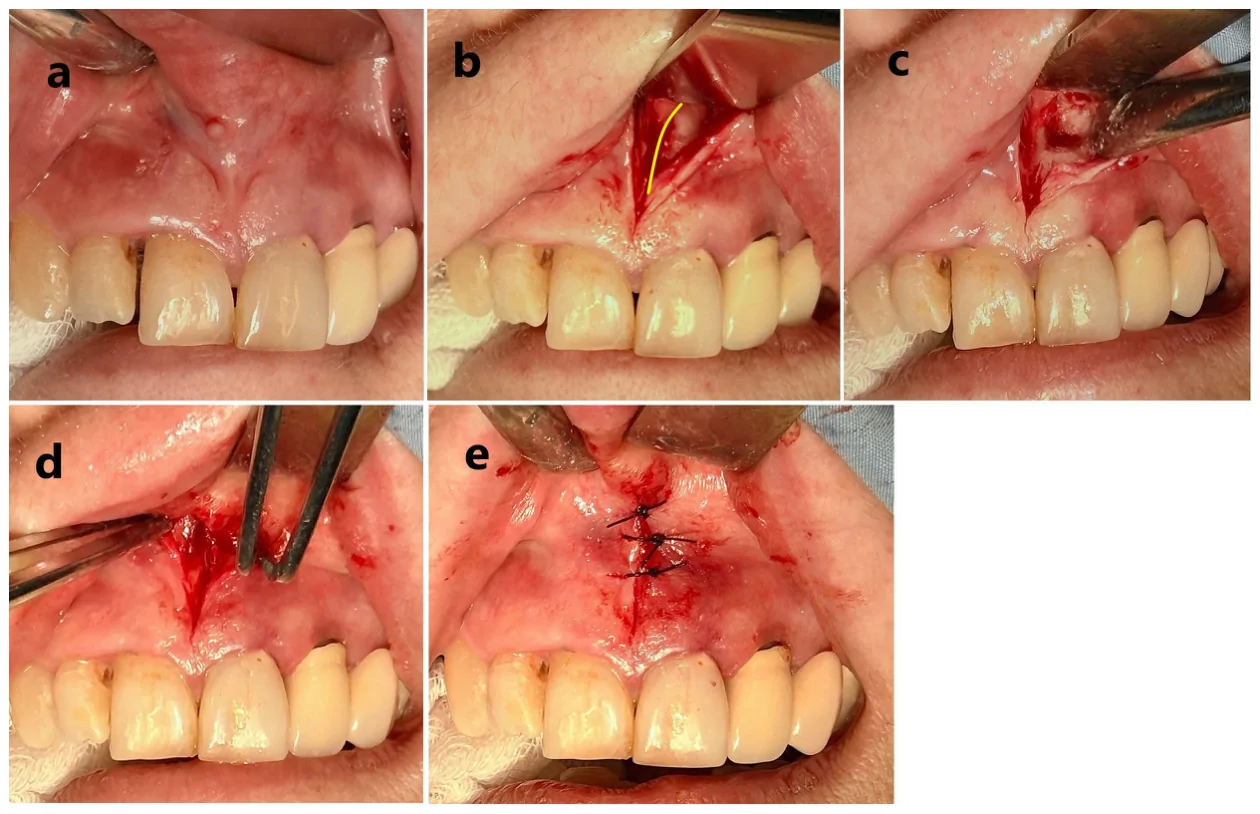
Intraoperative documentation of Case 2: (**a**) preoperative soft-tissue presentation; (**b**) retractor placement through the sagittal mucosal window while preserving the deep tissue layers (the yellow line depicts the second incision through the periosteal tissue); (**c**) reflected periosteal architecture exposing the cortical defect, with the lateral part of the tissue already retracted; (**d**) closure of the periosteum primary layer using a resorbable suture; (**e**) superficial mucosal-layer adaptation using a 4-0 pseudo-monofilament suture (Supramid; Serag-Wiessner, Naila, Germany).

**Figure 5:**
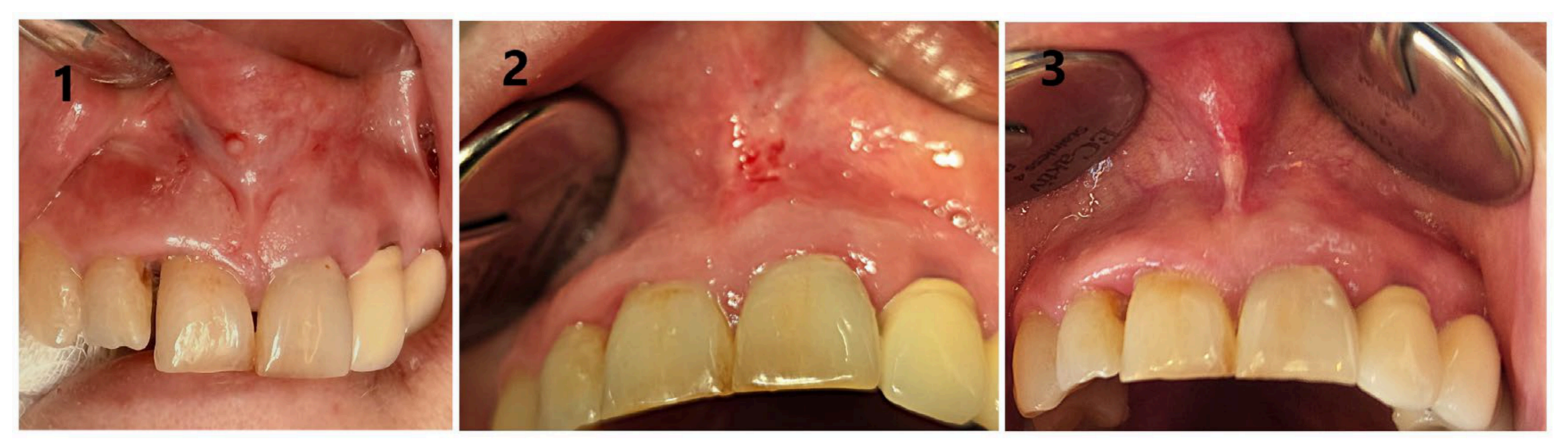
Clinical follow-up sequence of Case 2: (**1**) preoperative initial status; (**2**) soft-tissue healing evaluation on postoperative day 7 immediately following suture removal; (**3**) three-month postoperative clinical control showing stable marginal gingiva, a complete absence of recession, and optimal frenular integration.

A 60-year-old female patient presented to our oral surgery clinic requesting treatment for a persistent periapical lesion associated with tooth 21, with tobacco smoking documented as a behavioral risk factor. Following clinical assessments and radiographic diagnostics, endodontic retreatment alternatives were thoroughly discussed, and the decision was made to perform a surgical root-end intervention using the Transfrenular Single-Incision (TrSI) protocol. The baseline clinical condition of the anterior maxillary soft tissues was recorded (Figure 4a and Figure 5). The planned microsurgical procedure was explained in detail, and written informed consent was obtained from the patient.

Patient preparation and local antisepsis were performed analogously to Case 1 using a 0.12% chlorhexidine digluconate mouthwash (Gum Paroex; Sunstar, Schönau, Germany) under standard sterile draping, and local anesthesia was achieved via local infiltration of approximately 1.5 mL of 4% articaine with 1:200,000 adrenaline (Ultracain D-S; Septodont, Frankfurt am Main, Germany).

The micro-surgical phases were performed in strict accordance with the established TrSI protocol. A sharp sagittal midline mucosal incision was made within the labial frenulum using a conventional No. 15 scalpel blade (B. Braun; Melsungen, Germany), and following blunt lateral submucosal dissection with fine scissors, a Langenbeck retractor (Ustomed; Tuttlingen, Germany) was carefully placed through the mucosal window to facilitate clear visualization while leaving the deep tissue and periosteal layers completely intact (Figure 4b).

Once adequate space was established, a secondary vertical incision was made directly through the periosteum, with the exact incision direction depicted by the yellow line in Figure 4b. While the second incision was being performed, the scalpel blade was pressed down to the cortical plate, and the periosteal architecture was reflected laterally using a periosteal elevator. Inserting the Langenbeck retractor (Ustomed; Tuttlingen, Germany) further safely exposed the periapical bone defect (Figure 4c). Standard osteotomy with round bur (H 141; Komet Dental, Lemgo, Germany) and root-end resection (Lindemann bur; Komet Dental, Lemgo, Germany) were performed under continuous sterile saline irrigation. Retrograde cavity preparation was completed using a sonic scaler handpiece (SONICflex; KaVo Dental, Biberach, Germany), and the cavity subsequently sealed with a premixed bioceramic putty (NeoPUTTY; Avalon Biomed, Houston, TX, USA).

Wound closure was ensured using a synchronized, two-layer framework to ensure a tight bacterial seal. First, the deep periosteal layer was repositioned and safely closed using a 5-0 monofilament resorbable suture (Serafast; Serag-Wiessner, Naila, Germany) to restore periosteal integrity over the bone defect (Figure 4d), while the secondary superficial mucosal layer was sealed using a 4-0 pseudo-monofilament non-resorbable nylon suture (Supramid; Serag-Wiessner, Naila, Germany) to completely reconstruct the original anatomy of the labial frenulum (Figure 4e).

Postoperative pharmacology and home-care instructions matched the protocol described in Case 1, with the patient returning on the seventh postoperative day for follow-up and suture removal. Clinical evaluation immediately following suture removal demonstrated excellent soft-tissue healing kinetics and satisfactory primary closure (Figure 5), while the 3-month longitudinal postoperative control demonstrated complete soft-tissue rehabilitation, stable marginal gingiva, zero marginal recession, and optimal aesthetic integration of the reconstructed frenulum upon clinical examination (Figure 5).

The postoperative course was uneventful; at suture removal, the patient was pain-free, reporting only minor discomfort during chewing. By the 3-month follow-up, all symptoms had completely resolved, and the patient expressed complete satisfaction with the achieved functional and aesthetic results.

## 4. Discussion

Numerous clinical obstacles can cause orthograde endodontic treatments to fail, and complex anatomical configurations, such as apical ramifications, isthmuses, and lateral canals, alongside canal calcifications, obstructions, and iatrogenic protocol errors, frequently prevent adequate chemo-mechanical debridement and three-dimensional sealing. When these compounding factors result in persistent periapical pathology, PSI becomes the necessary therapeutic alternative [5].

In periapical surgery, hard-tissue regeneration is positively correlated with the quality of the soft-tissue healing cascade [6]. In an analysis of 242 endodontic surgeries, Verardi [7] concluded that periapical procedures carry an inherent risk of inducing gingival recession, with significant alterations in periodontal parameters typically observed within the first postoperative year. Consequently, selecting the appropriate flap design is a critical determinant during apicoectomies involving the anterior aesthetic zone, as it directly influences postoperative probing depths and clinical attachment loss [8,9].

Modern PSI has shifted its paradigm; it no longer focuses solely on eradicating the root apex and infected periapical tissue but instead prioritizes optimal soft-tissue management as a prerequisite for predictable osseous healing.

Grandi and Pacifici [3] delineated the various flap designs utilized to gain access to periapical defects, which are primarily categorized by the main incision configuration: sulcular, submarginal, or a combination of both.

Velvart and Peters [10] defined the ultimate goal of contemporary endodontic microsurgery as a recession-free and predictable healing process of the gingival architecture. Due to its prominent visibility during animation, the marginal gingiva of the maxillary central incisors is paramount for the “red aesthetics” of the smile. Beyond marginal recession, visible scar formation can severely compromise the cosmetic outcomes of conventional surgical approaches [11,12].

Extensive research has focused on intrasulcular incisions, submarginal incisions, and the papilla base flap [13,14,15,16,17,18,19,20]. Historically, these comparative investigations can be divided into three consensus groups: those demonstrating superior aesthetic outcomes for papilla-based incision, those favoring the intrasulcular approach, and those reporting no significant difference between the techniques.

A systematic review and meta-analysis [13] confirmed that intrasulcular incisions are significantly associated with an increased risk of marginal gingival recession. Furthermore, Velvart et al. [15] demonstrated that the conventional sulcular flap resulted in a significant loss of interproximal papilla height, leading to the development and advocacy of the papilla base incision to prevent interproximal space collapse or opening in aesthetically sensitive zones [16,17]. Conversely, while intrasulcular incisions and the PBI tend to produce less visible scarring, submarginal flaps are frequently associated with a higher incidence of keloid-like scar formation along the mucosal line [7,11].

In contrast, another cohort of studies has failed to identify any statistically significant differences regarding gingival recession among distinct incision lines. For example, Taschieri et al. [18] reported similar clinical outcomes in terms of papilla height variations and marginal tissue alterations across different designs, subsequently concluding in a separate study [20] that no single-incision technique demonstrated absolute superiority over the others. Additionally, a systematic review and meta-analysis from 2021 [9] corroborated these findings, leaving the debate open regarding the gold-standard option for the anterior aesthetic zone.

Given the lack of consensus and the controversial data surrounding optimal anterior flap designs, the authors introduced the Transfrenular Single-Incision (TrSI) flap. The classic single vertical incision is well-regarded for minimizing surgical trauma and eliminating muscular traction on the flap margins [3]. By completely bypassing the marginal and papillary gingiva, the TrSI flap leaves the periodontal attachment intact, thereby eliminating the risk of gingival recession, which is most beneficial when central incisors have a full restorative crown or aesthetic veneer facets (lenses).

However, a traditional single vertical incision presents a major clinical challenge, namely the risk of suturing directly over an active osseous defect, which is a known predisposing factor for wound dehiscence in oral surgery. The modification originally proposed by Eskici [4] addressed this limitation through a specialized two-layer closure mechanism that minimizes dehiscence risk. The TrSI flap not only builds upon this concept of a double-layer suture that is rooted in biomechanical principles; it also functions as an impermeable biological barrier, a vascular stabilizer, and a histological guide. Therefore, periosteal and mucosal suture lines are laterally offset from one another, thus effectively neutralizing micro-leakage and preventing wound breakdown.

Furthermore, the literature indicates that the reflection of both full-thickness and partial-thickness mucoperiosteal flaps triggers transient osteoclastic activity, leading to localized cortical bone loss [21]. In contrast, eliminating a marginal sulcular incision preserves the vascularity and vitality of the root-attached soft tissues [22]. The TrSI flap respects this physiological principle by confining periosteal elevation strictly to the apical region of the targeted tooth. Consequently, only the minimal surface area of bone required to perform the osteotomy is deperiostealized, while the peripheral cortical bone remains protected by intact, vascularized periosteum in all directions.

A separate modification of the single-incision approach, known as the Minimally Invasive Vertical Incision Subperiosteal Tunneling Technique (MIVIST), was introduced by Abella et al. [23] for PSI. However, the MIVIST protocol relies on additional vertical releasing incisions to facilitate tissue tunneling. In contrast, the TrSI flap achieves submucosal tunneling through a single, central sagittal incision. The delicate dissection of the labial frenulum fibers and the initiation of lateral tunneling directly through this connective tissue base represent a novel departure from both the classical single-incision flap and the MIVIST protocol. Thus, the anatomical repositioning and layer-by-layer suturing of these frenular fibers during primary closure serve as a key stabilizing element for the submucosal architecture.

No postoperative complication, such as wound breakdown or fistula formation, was observed in any of the clinical cases treated with the TrSI approach. Moreover, any postoperative scarring was minimal, with minor cicatrization remaining localized precisely along the natural sagittal border of the frenulum, rendering it practically invisible without high-power magnification. Therefore, the absolute absence of horizontal or oblique incisions through the mucosa or periosteum ensures that the regional blood supply is optimally preserved.

The primary limitation of the TrSI technique is its restrictive indication, as it is strictly limited to the maxillary central incisors. Although its application in the mandibular anterior region is anatomically analogous, it has not yet been clinically implemented by the authors for PSI involving central and lateral inferior incisors. Additionally, from a technical standpoint, the TrSI flap requires advanced microsurgical skills and has a steep learning curve. The relatively confined surgical window may also limit its utility in cases involving extensive cystic lesions or procedures requiring multi-tooth endodontic access.

A further aspect warranting research is the combination and implementation of surgical guides in apicoectomies, as studies support the thesis that utilizing surgical guides can be beneficial for improved soft-tissue healing [24].

## 5. Conclusions

The Transfrenular Single-Incision (TrSI) flap modification offers a highly predictable, minimally invasive approach for periapical surgery in the anterior maxilla. By confining the primary surgical entry point to the sagittal midline of the maxillary labial frenulum and utilizing a sub-mucosal tunneling framework, this technique preserves the marginal and papillary gingiva. Consequently, the risks of postoperative gingival recession and visible scar formation, which are frequently associated with conventional sulcular or submarginal flaps, are effectively minimized, yielding excellent “red aesthetics” and optimal soft-tissue healing.

Crucially, to date, no short- or long-term clinical complications have been observed in relation to the maxillary labial frenulum or upper-lip dynamics. The preservation of the submucosal architectural framework and the meticulous execution of the offset, two-layer closure effectively eliminated any risk of wound dehiscence, tissue scarring, or hypertrophic cicatrization. Furthermore, the patients exhibited no signs of restricted lip mobility, asymmetric smile animation, or sensory disturbances, thereby confirming the safety, predictability, and superior aesthetic biocompatibility of the TrSI flap modification for paraendodontic piezo-microsurgery in the anterior maxilla.

The primary limitation of the TrSI technique is its narrow indication spectrum, as its clinical application is restricted to the maxillary central incisors. Additionally, the localized surgical window demands advanced microsurgical skills from the clinician. Further long-term, prospective clinical trials involving larger sample sizes are warranted to quantitatively evaluate periodontal parameter changes, patient-reported outcome measures, and microscopic scar formation over extended follow-up intervals.

The reported findings are somewhat empirical due to the number of patients treated (*n* = 2). Thus, further research involving a larger patient cohort and a longer follow-up period is essential to establish the robustness of the TrSI flap. Additionally, statistical analyses and an evaluation of the long-term results are needed to compare this flap against other flap designs.

## Data Availability

The original contributions presented in this study are included in the article. Further inquiries can be directed to the corresponding author.

## References

[1] Mushtaq A., Alsanafi S., Elmsmari F., González J.A., Garcia-Font M., Abella Sans F., Afrashtehfar K.I., Abbott P.V. (2026). Effect of root canal filling techniques and materials on endodontic treatment outcomes: A systematic review and meta-analysis. Sci. Rep..

[2] Haxhia E., Ibrahim M., Bhagavatula P. (2021). Root-end Surgery or Nonsurgical Retreatment: Are There Differences in Long-term Outcome?. J. Endod..

[3] Grandi C., Pacifici L. (2009). The ratio in choosing access flap for surgical endodontics: A review. Oral Implantol..

[4] Eskici A. (1971). Eine neue Schnittführung bei Wurzelspitzenresektionen: A new incision method in apicoectomy. Dtsch. Zahnärztl. Z..

[5] Ferraro C., Cernera M., Abdellatif D., Galdi M., Esposito L., Spagnuolo G., Mancino D., Iandolo A. (2025). SEM Analysis and Pulp Tissue Dissolution Following Retrograde Preparation and Irrigation in Surgical Endodontics: A Novel Approach. Eur. Endod. J..

[6] Ng Y.L., Gulabivala K. (2023). Factors that influence the outcomes of surgical endodontic treatment. Int. Endod. J..

[7] Verardi S. (2012). Apical surgery may cause gingival recession. J. Evid. Based Dent. Pract..

[8] von Arx T., Vinzens-Majaniemi T., Bürgin W., Jensen S.S. (2007). Changes of periodontal parameters following apical surgery: A prospective clinical study of three incision techniques. Int. Endod. J..

[9] Jamal S., Gul M., Khan F.R., Ghafoor R. (2021). Effect of full sulcular versus papilla-sparing flap on periodontal parameters in periradicular surgeries: A systematic review and meta-analysis. J. Indian Soc. Periodontol..

[10] Velvart P., Peters C.I. (2005). Soft tissue management in endodontic surgery. J. Endod..

[11] Ahmed M.V., Rastogi S., Baad R.K., Gupta A.K., Nishad S.G., Bansal M., Kumar S., Oswal R., Mahendra P., Bhatnagar A. (2013). Comparative Study Between Two Flaps-Trapezoidal flap (TZF) and Ocshenbein-Leubke Flap (OLF) in Periapical Surgeries. J. Maxillofac. Oral Surg..

[12] Arx T.V. (2008). Scarring of gingiva and alveolar mucosa following apical surgery: Visual assessment after one year. Oral Surg..

[13] Alamoudi R.A., Alghamdi N.S., Alqahtani S.M., Alamoudi R.A.S., Baghlaf K. (2021). Gingival Recession after Surgical Endodontic Treatment and Quality of Life: A Systematic Review and Meta-analysis. Oral Health Prev. Dent..

[14] Castro-Calderón A., Toledano-Serrabona J., Sánchez-Torres A., Camps-Font O., Sánchez-Garcés M.Á., Gay-Escoda C. (2021). Influence of incision on periodontal parameters after apical surgery: A meta-analysis. Clin. Oral Investig..

[15] Velvart P., Ebner-Zimmermann U., Ebner J.P. (2004). Papilla healing following sulcular full thickness flap in endodontic surgery. Oral Surg. Oral Med. Oral Pathol. Oral Radiol. Endod..

[16] Velvart P., Ebner-Zimmermann U., Ebner J.P. (2004). Comparison of long-term papilla healing following sulcular full thickness flap and papilla base flap in endodontic surgery. Int. Endod. J..

[17] Velvart P., Ebner-Zimmermann U., Ebner J.P. (2003). Comparison of papilla healing following sulcular full-thickness flap and papilla base flap in endodontic surgery. Int. Endod. J..

[18] Taschieri S., Del Fabbro M., Francetti L., Perondi I., Corbella S. (2016). Does the Papilla Preservation Flap Technique Induce Soft Tissue Modifications over Time in Endodontic Surgery Procedures?. J. Endod..

[19] Albanyan H., Aksel H., Azim A.A. (2020). Soft and Hard Tissue Remodeling after Endodontic Microsurgery: A Cohort Study. J. Endod..

[20] Taschieri S., Corbella S., Del Fabbro M. (2014). Do gingival soft tissues benefit from the application of a papilla preservation flap technique in endodontic surgery?. J. Oral Maxillofac. Surg..

[21] Fickl S., Kebschull M., Schupbach P., Zuhr O., Schlagenhauf U., Hürzeler M.B. (2011). Bone loss after full-thickness and partial-thickness flap elevation. J. Clin. Periodontol..

[22] Harrison J.W., Jurosky K.A. (1991). Wound healing in the tissues of the periodontium following periradicular surgery. I. The incisional wound. J. Endod..

[23] Abella Sans F., Barragán Montes J., Zbozen T., Suresh N., Dharmarajan L., Dummer P.M.H., Nagendrababu V. (2025). Minimally Invasive Vertical Incision Subperiosteal Tunnelling Technique for Targeted Endodontic Surgery: Technical Overview and a Case Report. Int. Endod. J..

[24] Sánchez-Herrera G., Facchera M., Palma-Carrió C., Pérez-Leal M. (2025). Approaches in apical microsurgery: Conventional vs. guided. A systematic review. Oral Maxillofac. Surg..

